# COVID-19 Infection among Nursing Students in Spain: The Risk Perception, Perceived Risk Factors, Coping Style, Preventive Knowledge of the Disease and Sense of Coherence as Psychological Predictor Variables: A Cross Sectional Survey

**DOI:** 10.3390/nursrep12030066

**Published:** 2022-09-16

**Authors:** Diego Serrano-Gómez, Verónica Velasco-González, Ana Rosa Alconero-Camarero, José Rafael González-López, Montserrat Antonín-Martín, Alicia Borras-Santos, Montserrat Edo-Gual, Vicente Gea-Caballero, José L. Gómez-Urquiza, Alfonso Meneses-Monroy, Montserrat Montaña-Peironcely, Carmen Sarabia-Cobo

**Affiliations:** 1 Faculty of Health Sciences, Universidad de Burgos, 09001 Burgos, Spain; 2 Nursing Care Research Group (GICE), Department of Nursing, Faculty of Nursing, Universidad de Valladolid, 47005 Valladolid, Spain; 3 Faculty of Nursing, Universidad de Cantabria, IDIVAL Nursing Research Group, 39008 Santander, Spain; 4 Department of Nursing, Faculty of Nursing, Physiotherapy and Podiatry, Universidad de Sevilla, 41009 Sevilla, Spain; 5 Escola Universitària d’Infermeria, Escoles Universitàries Gimbernat, Universitat Autónoma de Barcelona, 08174 Barcelona, Spain; 6 Faculty of Health, Universidad Internacional de Valencia, 46002 Valencia, Spain; 7 Faculty of Health Sciences, Universidad de Granada, 18071 Granada, Spain; 8 Faculty of Nursing, Physiotherapy and Podiatry, Universidad Complutense de University of Madrid, 28040 Madrid, Spain; 9 Parc Taulí Hospital Universitari, Grup Recerca d’Infermeria, Institut d’Investigació i Innovació Parc Taulí (I3PT), Universitat Autónoma de Barcelona, 08208 Sabadell, Spain

**Keywords:** nursing students, coping behaviors, COVID-19, salutogenesis, risk factors, nurses

## Abstract

The exploration of patterns of health beliefs about COVID-19 among nursing students may be beneficial to identify behaviors, attitudes and knowledge about contagion risk. We sought to analyze the variables of risk perception, perceived risk factors, coping style, sense of coherence and knowledge of preventive measures as possible predictors of having suffered from COVID-19. Participants were nursing students from 13 universities in Spain. Sociodemographic and health variables were collected. To test the independent variables, the Perception Risk Coping Knowledge (PRCK-COVID-19) scale was created and validated because there was no specific survey for young people adapted to the pandemic situation of COVID-19. It was validated with adequate psychometric properties. A total of 1562 students (87.5% female, mean age 21.5 ± 5.7 years) responded. The high perception of the risk of contagion, the high level of knowledge and a coping style focused on the situation were notable. Significant differences by gender were found in the coping styles, problem-focused, avoidance and knowledge scales, with women scoring higher in all categories. The multiple regression analysis was significant (F = 3.68; *p* < 0.001). The predictor variables were the coping styles subscale search for support and the intrinsic and extrinsic perceived risk factors. Our model predicts that nursing students with a social support-based coping style are at a higher risk of becoming infected with COVID-19, based on their own health belief model.

## 1. Introduction

It is evident that the recommendations to the population for the adoption of safety measures against COVID-19 have failed [1]. Classified as a worldwide pandemic, COVID-19 has affected more than 83 million people worldwide (1,893,502 in Spain) and caused more than 1.8 million deaths (50,000 in Spain) [2,3]. Recent research shows that health systems have allocated more resources to hospital and clinical care than to community care [4]. Experts warn that the key is to prevent the onset of the disease and not just treat it when its spread cannot be contained [5]. Hence, community and public health strategies are keys in prevention efforts.

These strategies are in line with the Health Belief Model [6,7] (Rosenstock, 1966, 1976), by which people, in general, present greater illusory optimism and a lower perception of risk, which are well-studied facts in processes such as adherence to treatment or prevention of risky behaviors (drugs, sexual practices, etc.) [8]. Rosenstock’s Health Belief Model (HBM) is “a theoretical model concerned with health decision-making. The model attempts to explain the conditions under which a person will engage in individual health behaviors, such as preventative screenings or seeking treatment for a health condition” [6,7]. The model is based on the assumption that people’s willingness to change their health behaviors primarily comes from their health perceptions. Individual beliefs about health and health conditions play a role in determining health-related behaviors. The psychological and cognitive processes underlying the Health Belief Models indicate that psychological constructs such as risk perception, coping style and knowledge perception are keys in the adoption of preventive measures against certain communicable diseases [9]. Thus, recent studies on COVID-19 focus on these psychological constructs [10]. Among the health models, one model that stands out in the field of public health and health promotion is the salutogenic model [11], which relates one’s approach to stressful situations (such as the COVID-19 pandemic) to the individual’s capacity for self-management of such situations [12]. This model develops concepts such as sense of coherence (SOC), which is directly related to the ability to employ cognitive, affective and instrumental strategies to better cope with stress [11].

Studies relating other psychological constructs besides SOC to the risk of acquisition of COVID-19 are unknown. Our study focused on nursing students (NS). Their lack of clinical experience, combined with their knowledge of preventive measures, may be valuable indicators for developing a predictive model relating psychological constructs such as SOC and coping styles to the likelihood of contracting the virus. This study constitutes an important aid in designing health education strategies in two manners: one, to inform the teachers of NS who perform their clinical placements, and two, based on general policies, since young people under 30 years of age seem to have a lower perception of risk in relation to the transmission of COVID-19 [13]. Our hypothesis considers that it is possible to predict the risk of contagion in nursing students, based on knowledge of psychological variables that, according to the literature, can act as mediators in this sense. These variables would have to relate to knowledge and coping strategies in the face of contagion, such as those we have described.

The main objective was to analyze the variables of risk perception, perceived risk factors, coping styles, SOC and knowledge of preventive measures among NS as possible mediating and predictor variables for contracting COVID-19.

## 2. Materials and Methods

### 2.1. Design

An observational single-group cross-sectional study was conducted. The participants were nursing students of 13 Spanish universities in all years of the degree. In Spain, the degree in nursing has a duration of four years and the students carry out clinical placements in health institutions from the second year onwards. Data collection was anonymous and did not entail any academic benefit for the students. Students were informed that participation was voluntary and that their involvement in the study would not affect their grade. We followed the Strengthening the Reporting of Observational Studies in Epidemiology (STROBE) [14]. All students received the instructions about the study by email with an information sheet and informed consent agreement. It was explained to them that their participation was anonymous, confidential and had no repercussions for the results.

### 2.2. Sample Size

Purposeful sampling methods were used. The study universe comprised a total of 7479 students. The sample size was calculated taking into account the statistical formula for prevalence of a known universe using the Grammo program (https://www.imim.es/ofertadeserveis/software-public/granmo/, accessed on 1 September 2020). It was increased by 20% to account for possible losses. Obtaining a minimum sample size of 366 students was thus sufficient to estimate a representative population mean, with 95% confidence and a margin of error of 5.

### 2.3. Variables and Instruments

Sociodemographic variables were collected, including age, gender, place of residence (rural/urban), course, chronic diseases, number of cohabitants during confinement and smoking habits.

A series of COVID-19 variables were also considered (questions with yes/no responses): Have you had COVID-19? Has anyone in your close environment suffered from the virus? Has anyone in your close environment died from COVID-19?

Having suffered from the disease was considered the dependent variable (DV).

The following independent variables were collected:

- Sense of coherence was evaluated using the Orientation to Life Questionnaire—13 Items (OLQ-13 or SOC-13) [15]. This instrument measures a global personality orientation that facilitates adaptive problem solving in stressful situations. The 13-item questionnaire also measures the dimensions of understandability (5 items), manageability (4 items) and meaningfulness (4 items). The scores express the strength of the person’s SOC; the higher the score, the greater the strength. The answers offer a continuum of degree from minus to plus in 7 response options on a Likert scale from 1 to 7 (“never” “rarely” to “very often” or “always”), both in the positive and negative dimensions of the question. The OLQ-13 scale presents suitable internal consistency, with a Cronbach’s alpha between 0.70 and 0.92 [16,17], and retains the same psychometric qualities as the original 29-item version. In this study, the internal consistency of the items was analyzed using Cronbach’s alpha for the total scale (0.71) and for the comprehensibility (0.81), manageability (0.79) and significance (0.71) subscales.

For the assessment of risk perception, perceived risk factors and coping styles in the face of COVID-19, an ad hoc survey was designed by a panel of 6 experts based on the literature [18,19,20]. This survey, called the Perception. Risk. Coping. Knowledge (PRCK-COVID-19) scale, was created and validated because there was no specific survey for young people adapted to the pandemic situation of COVID-19. It was validated on a sample of 30 students, with adequate psychometric properties. The scales have been validated, piloted and created for the Spanish population. This survey consists of four scales:

- Perceived risk scale (3 items). The degree of agreement was shown on an LS (0, none and 10, maximum risk). The maximum score is 30 points, indicating that the higher the score, the higher the perceived risk of COVID-19 infection. Factor analysis identified a one-factor structure (Cronbach’s alpha = 0.735).
- Perceived risk factors scale (16 items). The degree of agreement according to an LS was shown (from 1, strongly disagree to 5, strongly agree). The factor analysis identified a two-factor structure (total Cronbach’s alpha = 0.781; FR1 alpha = 0.721; FR2 alpha = 0.841). The two factors identified correspond to risk factors perceived as external or dependent on the environment (9 items, extrinsic factor or FR1) or as personal factors that depend on their own behavior (7 items, intrinsic factor or FR2). The higher the score, the greater the weight of one risk factor over the other. The intrinsic factors are desirable because they refer to “the things I can do to protect myself”, whereas the extrinsic factors refer to the inevitability of the disease and “factors that are beyond my control and for which I can do nothing” [21].
- Coping styles scale in the face of contagion (19 items). This scale gathered the degree of agreement according to an LS (from 1, strongly disagree to 5, strongly agree). Eight items are reversed. Factor analysis identified a three-factor structure (total Cronbach’s alpha = 0.889). The three factors identified correspond to three coping styles when faced with COVID-19: EA1, reality-focused (7 items); EA2, avoidance (7 items); and EA3, support-seeking (5 items). The higher the score, the greater the weight of one risk factor over the other. Of the coping styles that coincide with the literature, reality-focused (greater self-efficacy) and support-seeking (5 items) are preferred [22].
- Preventive knowledge of COVID-19 scale (19 items). The degree of agreement was shown (from 1, strongly disagree to 5, strongly agree). The factor analysis identified a single factor structure called knowledge (Cronbach’s alpha = 0.57). Reverse items are included in this scale. The maximum score is 60 points; a score range from 50 to 60 points indicates high knowledge, and lower scores indicate less knowledge.

### 2.4. Procedure

All variables were integrated into an online survey using Google Forms that was sent to all participating universities for dissemination, for convenience, through mailing lists, between November 2020 and January 2021. Multiple responses were avoided with the response identification protocol. Completing the questionnaire took an average of 6 min. The students only completed the questionnaires indicated in this work.

### 2.5. Statistical Analysis

IBM SPSS Statistics 22 was used for the statistical analysis. A bilateral contrast and a confidence level of 95% were adopted. To analyze possible missing values, we used the EM method (expected maximization). A descriptive analysis was performed for all the variables studied (means and SD for quantitative variables and percentages for qualitative variables). Comparisons were made between the categories defined by the independent variables for all the scales evaluated (SOC, coping styles, risk factors and knowledge) by means of Student’s *t*-test for independent samples. A bivariate correlation analysis was performed between all the variables using Pearson’s r test. A forward stepwise multiple regression analysis was calculated using the dependent variable of having suffered from COVID-19 and the predictor variables of different SOC scales, risk perception, risk factors, coping styles and knowledge.

### 2.6. Ethics

The researchers had no conflicts of interest. Ethical approval was obtained from the Research Ethics Committee of the University of Cantabria, Spain (CE Proyectos 13/2020). The treatment of the data guaranteed their confidentiality and their exclusive use for this project, respecting the legislation in force. At the beginning of the questionnaire, there was a specific box for giving consent to participate.

## 3. Results

### 3.1. Descriptive Analysis of the Sample

A total of 1562 people responded (response rate = 20.88%). Women accounted for 87.5% of the sample. The mean age was 21.5 ± 5.7 years. Up to 76.8% resided in urban areas. Participation of NS was equal for all four academic years. Overall, 67.9% did not suffer from any chronic disease, and 86.3% were non-smokers. In relation to COVID-19, only 9% had suffered from the virus, confirmed with a PCR test. For 52.8%, someone in their environment had suffered from the disease and of these, 6.9% died from it (Table 1).

### 3.2. Perceived Risk, Risk Factors and Preventive Knowledge about COVID-19

The students analyzed showed a medium-high perception of risk of COVID-19 infection (67.2%), with a greater weight of extrinsic factors (FR1, 75.6%) than intrinsic factors (FR2, 56.9%). Those who stated that someone in their close environment had suffered from COVID-19 scored significantly higher. Those who had experienced COVID-19 scored significantly higher on FR1 (extrinsic), and those who had not suffered COVID-19 scored higher on FR2 (intrinsic). Intrinsic factors scored differently depending on the academic year. The score obtained on the knowledge scale, which reached a high level in the general population (54.25 +/− 4.95 out of 60), was significantly higher in women and in those in more senior years (Table 2).

### 3.3. Coping Styles in the Face of COVID-19

EA1 (reality-focused) coping styles, which acquired greater weight in the sample of students analyzed, scored significantly higher among females. AE2 (avoidance), which ranked last when analyzing the entire sample, scored significantly higher among females, first-year students and those who reported that someone in their close environment had suffered or died from COVID-19. AE3 (seeking support) scored significantly higher among fourth-year students and those who reported that they or someone close to them had suffered from COVID-19 (Table 3). Of the three styles, only for EA3 did those who had experienced COVID-19 score significantly higher.

### 3.4. Sense of Coherence towards COVID-19

The mean total SOC for the entire sample was 52.77 ± 6.71 points (out of 91), with the relative order of the dimensions, according to their percentage of each total, being understandability, manageability and meaningfulness. Females and first-year students scored significantly higher for the total SOC and in the understandability and meaningfulness dimensions. Males scored significantly higher in the manageability dimension. Those who stated that someone in their environment had died from COVID-19 scored significantly higher in the total SOC and the manageability dimension (Table 4).

### 3.5. Predictive Factors of Having Suffered from COVID-19

The correlational analysis indicated a significant association between the variable of having suffered from COVID-19 and all the SOC subscales (total r = −0.23, *p* < 0.001; comprehensibility r = −0.58, *p* < 0.001; manageability r = −0.21, *p* < 0.001; significance r = −0.22, *p* < 0.001), the risk perception scale (r = −0.47, *p* < 0.001), the risk factor subscales (FR1 r = −0.84, *p* < 0.001; FR2 r = −0.41, *p* < 0.001), the coping style subscales (EA1 r = −0.57, *p* < 0.001; EA2 r = −0.61, *p* < 0.001; EA3 r = −0.84, *p* < 0.001) and knowledge (r = −0.25, *p* < 0.001).

A forward stepwise multiple regression analysis was performed using having suffered from COVID-19 as the dependent variable and the different SOC scales, risk perception, risk factors, coping style and knowledge as predictor variables. The model was significant (F = 3.68; *p* < 0.001) and managed to explain 15% of the variance in the criterion variable (suffering from COVID-19) by means of the predictor variables EA3, FR1 and FR2. The subscale EA3 (support-seeking) is the most relevant predictor (beta = −0.12; *p* < 0.001), explaining 8% of the variance of the criterion variable, followed by FR1 (extrinsic factors) (beta = 0.07; *p* = 0.008) and FR2 (intrinsic factors) (beta = 0.06; *p* < 0.001) (Table 5).

## 4. Discussion

*Geographical context of the pandemic in the country of study.*

Spain is a country that has stood out throughout the pandemic considering two aspects in the management executed by the authorities. First, control has been exercised from the central government, but each region of the country has its own competencies in health legislation. This means that although a state of alarm was decreed throughout the national territory (with the mandatory confinement of the entire population between March and June 2020, limitation of mobility, control of capacity in premises, mandatory use of a mask indoors, etc.), each region, as of September 2020, has implemented its own measures, which could not contradict State regulations, but which sometimes differed from one area of the country to another. This situation, similar to other countries such as Italy and Portugal, led to an unequal approach to the control of the pandemic, with large differences in contagion and control. Shelling the comparative aspects of the sample by country zone exceeds the objectives of this study, but that is why it is representative of most regions of the country. It is usual in this type of study to carry out an extensive data collection (longitudinal and cross-sectional study design) and with a sample that is as geographically heterogeneous as possible. The second important milestone in Spain was the premature vaccination campaign (December 2020) compared to other European countries, with a high vaccination rate throughout the national territory. However, this fact comes after the data collection of this study, so even in spite of its relevance (in terms of the explanation of risk perception, coping styles and sense of coherence in the face of health behaviors such as getting vaccinated), it is not worth discussing here.

### 4.1. Descriptive Analysis of the Sample

Risk perception makes it possible to assess why people do or do not take measures to protect themselves from external threats [23]; therefore, it would be desirable for the scores to be proportionally higher in the intrinsic subscale (“I can control my behavior to avoid risk factors”). In our study, we found that the extrinsic risk factor (FR1) was the highest in the sample. This variable is related to the disease as a risk (linked to the inevitability of the disease and “factors that are alien to me and over which I have no influence”). Our results could be justified in part by the “inevitability” of the disease, transmitted through the media and the lack of reliable data in the face of an unknown disease that generates fear, stress and uncertainty [24].

Both subscales of risk perception factors revealed medium-high values, in agreement with those obtained by other authors in Belgian NS populations [25], Saudi populations [26], German populations [20], Pakistani populations [23] and Spanish populations [27]. As in other professions, being in higher years of study [26] is associated with an increased perception of risk. Surprisingly, in contrast to the results obtained by others [20,23,26,27], gender does not seem to influence risk perception, which may be due to the low male representation in our sample. Having close experiences with COVID-19 reduces the perception of risk, in contrast with previous findings [25,27], although in these former studies, the “experiences” involve professional patient care, and in our study, the experiences are more related to the family or social environment.

The population analyzed showed high knowledge of prevention, which coincides with other studies conducted among nursing students [25,28] and medical students [29]. This is probably due to the fact that in the latter, data collection was carried out at the beginning of the pandemic, when numerous studies were being conducted [24,27]. In line with other authors, women [26,28] and students in higher years [26] who carried out their clinical practices during the pandemic showed greater knowledge. Unlike what was observed for risk factors, in our sample, knowledge was not affected by experiences related to COVID-19.

In relation to the coping styles scale, several papers have been published that use different scales to study the coping strategies used by the general population [20,23,30] or NS in particular [29,30] in the face of the pandemic. In line with our findings, other studies [31,32] have also found that situation-focused coping strategies are the most employed by students to face COVID-19, and they are more employed by females [20,23,31]. In our study, significant differences were found between men and women for the situation-centered and avoidance coping styles, with higher scores among women in both. It is also noteworthy that EA3 (support-seeking) yielded statistically significant differences between people who have suffered from the disease and those who have not, with higher scores in the former [33].

The most significant findings in SOC values revealed medium values on the total scale and on the three subscales, which is in line with similar studies [34,35]. Women scored significantly higher on the comprehensibility and meaningfulness subscales, while men scored significantly higher on the manageability subscales, consistent with a former study [36]. Men present more practical and applied coping values than women, who find more meaning and understanding in what is happening, finding a meaning that allows them to deal better with stressful situations [37]. However, despite the significant values in the SOC variable, this has not played any relevant role in the predictive model, unlike other studies [38,39]. It is likely that this may be due to the fact that the variables of the coping styles and risk perception scales have displaced this other scale when it comes to coping with the situation, a fact that is corroborated by studies on prevention and health promotion [40,41].

### 4.2. Predictive Model

From the perspective of psychological variables related to prevention and a model of health beliefs, we found three variables to predict having suffered from the disease: the coping styles subscale, *search for support*, and the intrinsic and extrinsic perceived risk factors, which explain 15% of the variance. These results seem to indicate that people who, according to our support-seeking subscale, seek support in the opinion of experts, the media, third parties or government measures, contracted COVID-19. Relying on changing and contradictory information from authorities could justify this coping style, as has been partially suggested by other studies [13,42]. Those with an extrinsic risk factor present passive behaviors and attitudes, focused on the inevitability of the situation versus those with an intrinsic style focused on their own ability to protect themselves and take action. Both styles could be related to internal and external locus of control, as suggested by previous authors [43]. Interestingly, both are predictor variables, although to a lesser extent. Whereas the extrinsic factor is contemplated in other studies, as it favors adopting a passive and non-preventive attitude towards contagion [44], the intrinsic factor is not so easy to explain, suggesting the need for further research.

This study has several limitations. It is important to note that a sampling selection was used, and although it was intended to be a representative sample, generalization of the findings should be considered with caution. It should also be noted that although the questionnaire used was validated with adequate psychometric properties, it is necessary to confirm these findings on a larger sample. Analysis of long-term maintenance of acquired knowledge and reinforced attitudes may be a future line of research. It would be interesting to extend the sample to the same universities as well as to other students of health sciences, such as medicine. We must also be cautious with the generalization of the results in these types of studies due to the design (cross-sectional study). Another important limitation is that all the measures were self-report questionnaires so that the subjective value of responding is conditioned by the circumstances surrounding the person.

## 5. Conclusions

Our predictive model allows us to predict that NS with a coping style based on social support and a perception of high intrinsic and extrinsic risk factors present a greater risk of contracting COVID-19, according to their own model of health beliefs. Women presented greater knowledge of preventive measures and a more situation-focused coping style than men.

Female students in their final years, with more knowledge and experience in clinical practice, also presented more knowledge, a more extrinsic risk perception and a more situation-focused and less avoidant coping style than younger students. In terms of SOC, females scored significantly higher in total SOC and in the comprehensibility and meaningfulness dimensions, whereas males scored significantly higher in the manageability dimension.

Our study provides a model of health beliefs that can be considered when focusing on the preventive measures to be implemented among NS who must undertake their clinical practices.

Investing in training and educating in a health belief model that addresses psychological variables such as risk perception, coping styles and sense of coherence may have important benefits for career and internship curriculum design.

## Figures and Tables

**Table 1:** Sociodemographic variables of the sample and questions related to COVID-19.

		N	%
Gender ^†^			
	Female	1366	87.5%
	Male	193	12.4%
Age ^†^		M 21.5	DE 5.7
Place of residence ^†^	Rural	351	22.5%
	Urban	1200	76.8%
Academic year ^†^			
	1st	432	27.7%
	2nd	413	26.4%
	3rd	346	22.2%
	4th	355	22.7%
Do you have any of the following chronic diseases?	No	1060	67.9%
	Allergy	224	14.3%
	Asthma	109	7.0%
	Diabetes	11	0.7%
	Hypertension	7	0.4%
	Obesity	22	1.4%
	Others *	129	8.3%
Do you currently smoke? ^†^			
	No	1348	86.3%
	Yes	204	13.1%
Have you had COVID-19 (confirmed by PCR and/or serology)? ^†^			
	No	1406	90.0%
	Yes	140	9.0%
Has anyone in your close environment suffered from the virus? ^†^			
	No	726	46.5%
	Yes	824	52.8%
Has anyone close to you died from COVID-19? ^†^			
	No	1443	92.4%
	Yes	108	6.9%

^†^ Variable not answered by the totality of the sample. * Other diseases such as asthma, hypertension, hypothyroidism, diabetes, hyperlipidemia, cancer, bronchitis, etc.

**Table 2:** Gender variables, academic year and having suffered COVID-19 by risk perception, risk factors and knowledge subscales (descriptive and differential analysis).

Variable	N	Total Perceived Risk (Range 0–30)	FR1 (Range 9–45)	FR2 (Range 7–35)	Knowledge (Range 50–60)
		M/SD	M/SD	M/SD	M/SD
Total	1559	20.15 (4.27)	34.00 (4.04)	19.93 (2.88)	54.25 (4.95)
Gender					
Female	1366	20.17 (4.25)	34.05 (4.06)	19.97 (2.88)	54.44 ** (4.88)
Male	193	20.03 (4.45)	33.63 (3.94)	19.58 (2.86)	52.91 (5.23)
Academic year					
1st	432	20 (4.32)	33.71 (4.25)	19.89 (2.92) *	53.62 ** (5.38)
2nd	413	20.2 (4.20)	33.98 (3.85)	20.25 (2.80) *	54.15 (4.70)
3rd	346	20.05 (4.29)	33.88 (3.99)	19.49 (2.89) *	54.08 (5.28)
4th	355	20.34 (4.30)	34.49 (4.06)	19.89 (2.86) *	55.25 (4.16)
Have you had COVID-19 (confirmed by PCR and/or serology)?					
Yes	140	19.99 (4.92)	34.07 (3.95) *	19.43 (3.18) *	54.55 (4.78)
No	1406	20.16 (4.21)	33.20 (4.82)	19.97 (2.84)	54.24 (4.97)
Has anyone close to you died from COVID-19?					
Yes	108	20.15 (3.98)	33.78 (3.83)	20.02 (2.39)	54.68 (4.24)
No	1443	20.14 (4.30)	34.01 (4.05)	19.92 (2.91)	54.21 (5.00)
Has anyone in your close environment suffered from COVID-19?					
Yes	726	20.58 (4.25) **	34.11 (4.03)	19.93 (2.74)	54.29 (4.77)
No	824	19.65 (4.26)	33.88 (4.06)	19.92 (3.02)	54.19 (5.15)

* *p* value < 0.05. ** *p* value < 0.001. FR1 = extrinsic risk factors. FR2 = intrinsic risk factors.

**Table 3:** COVID-19 coping styles. EA1 (situation-focused coping style); EA2 (avoidance coping style); EA3 (support-seeking coping style).

Variable	N	EA1 (Range 7–35)	EA2 (Range 7–35)	EA3 (Range 5–25)
		M/SD	M/SD	M/SD
Total	1532	27.79 (4.15)	18.27 (5.48)	17.16 (3.96)
Gender				
Female	1366	27.92 (4.12) **	18.51(5.44) **	17.18 (3.97)
Male	193	26.8 (4.25)	16.69 (5.46)	17.04 (3.89)
Academic year				
1st	432	27.53 (4.47)	19.16 (5.69) **	16.62 (4.15)
2nd	413	27.63 (3.97)	18.58 (5.47)	17.24 (3.68)
3rd	346	27.73 (4.27)	17.65 (5.27)	16.96 (4.12)
4th	355	28.32 (3.81) *	17.47 (5.31)	17.93 (3.78) **
Have you had COVID-19 yourself (confirmed by PCR and/or serology)?				
Yes	140	28.22 (4.02)	18.60 (5.60)	18.24 (3.9) **
No	1406	27.74 (4.16)	18.23 (5.46)	17.06 (3.95)
Has anyone close to you died from COVID-19?				
Yes	106	28.05 (3.75)	19.58 (5.92) *	17.74 (4.40)
No	1423	27.76 (4.18)	18.17 (5.43)	17.11 (3.92)
Has anyone in your close environment suffered from COVID-19?				
Yes	814	27.70 (4.13)	18.64 (5.57) **	17.41 (3.93) *
No	718	27.88 (4.17)	17.86 (5.35)	16.90 (3.97)

* *p* value < 0.05. ** *p* value < 0.001. EA1 = situation-focused; EA2 = avoidance; EA3 = support-seeking.

**Table 4:** Sense of coherence (SOC) versus COVID-19.

Variable	N	SOC Total (Range 13–91)	SOC1 (Range 5–35)	SOC2 (Range 4–28)	SOC3 (Range 4–28)
		M/SD	M/SD	M/SD	M/SD
Total	1520	52.77 (6.71)	16.58 (3.63)	14.39 (2.67)	17.55 (2.62)
Gender					
Female	1366	52.94 (6.67) *	16.67 (3.58) *	14.34 (2.65)	17.64 (2.59) **
Male	193	51.60 (6.91)	16.04 (3.97)	14.78 (2.76) *	16.90 (2.71)
Academic year					
1st	432	54.09 (6.4) **	17.24 (3.36) **	14.64 (2.71)	17.82 (2.62) *
2nd	413	52.84 (7)	16.72 (3.71)	14.25 (2.77)	17.64 (2.64)
3rd	346	52.37 (6.37)	16.37 (3.66)	14.35 (2.44)	17.42 (2.44)
4th	355	51.56 (6.8)	15.89 (3.67)	14.31 (2.69)	17.23 (2.72)
Have you had it (confirmed by PCR and/or serology)?					
Yes	140	52.8 (6.69)	16.59 (3.06)	14.54 (2.9)	17.48 (2.66)
No	1406	52.76 (6.68)	16.57 (3.69)	14.38 (2.64)	17.56 (2.6)
Has anyone close to you died from COVID-19?					
Yes	105	54.66 (6.44) *	17.12 (3.42)	14.96 (2.84) *	17.92 (2.33)
No	1412	52.62 (6.72)	16.53 (3.65)	14.35 (2.65)	17.52 (2.64)
Has anyone in your close environment suffered from COVID-19?					
Yes	802	52.95 (6.76)	16.72 (3.59)	14.39 (2.62)	17.59 (2.56)
No	714	52.54 (6.66)	16.42 (3.67)	14.39 (2.69)	17.50 (2.68)

* *p* value < 0.05. ** *p* value < 0.001. SOC1 (comprehensibility); SOC2 (manageability); SOC3 (significance).

**Table 5:** Multiple regression analysis.

Predictors	Increase in R^2^	Increase in Adjusted R^2^	B	Standard Error	Beta	t	Sig.
EA3	0.8	0.8	−0.68	0.10	−0.12	−6.64	0.000
FR1	0.04	0.04	−0.22	0.08	0.07	−2.63	0.008
FR2	0.03	0.04	0.06	0.28	0.06	2.65	0.000

Dependent variable: having suffered from COVID-19. EA3 = support-seeking; FR1= extrinsic risk factors; FR2 = intrinsic risk factors. R^2^ total for the model = 0.15; R^2^ total model adjustment = 0.15 (F = 3.68; *p* < 0.001).

## Data Availability

The data presented in this study are available on request from the corresponding author.
